# Evaluation of an electronic diary for improvement of adherence to interferon beta-1b in patients with multiple sclerosis: design and baseline results of an observational cohort study

**DOI:** 10.1186/1471-2377-13-117

**Published:** 2013-09-06

**Authors:** Uwe K Zettl, Ulrike Bauer-Steinhusen, Thomas Glaser, Klaus Hechenbichler, Volker Limmroth

**Affiliations:** 1 Klinik und Poliklinik für Neurologie, Universitätsmedizin Rostock, Rostock, Germany; 2 Medizin Neurologie, Immunologie & Ophthalmologie, Bayer Vital GmbH, Leverkusen, Germany; 3 Institute Dr. Schauerte, Oberhaching, Germany; 4 Klinik für Neurologie, Städtisches Klinikum Köln-Merheim, Köln, Germany

**Keywords:** Adherence, Interferon beta-1b, Non-interventional study, Electronic diary, Multiple sclerosis, Immunomodulation

## Abstract

**Background:**

Multiple sclerosis is a chronic, incurable, demyelinating disease that requires long-term treatment. Rates of non-adherence to prescribed therapy of up to 50% have been reported for chronic diseases. Strategies to improve treatment adherence are therefore of the utmost importance. This study will evaluate the effect of using electronic and paper diaries on treatment adherence to interferon beta-1b in patients with a first clinical isolated syndrome (CIS) or relapsing-remitting multiple sclerosis (RRMS). Here we report on the study design and results of baseline assessments.

**Methods:**

Patients were recruited into a prospective national multicenter cohort study for an observational period of 2 years. At the start of the study, patients opted to use a digital (DiD) or paper diary (PD) to document self-administered injections of interferon beta-1b. Adherence to treatment will be assessed on the dropout rate at the end of the observation period and on the regularity of injections every other day at 6-month intervals. Patient-related health outcomes will also be evaluated.

**Results:**

700 patients with a mean age of 38.3 (SD 10.3) years and a mean duration of disease since diagnosis of 3.6 (SD 5.9) years were enrolled. 383 patients opted for the digital diary, 192 of which included an injection reminder. Significantly more male than female patients opted for the DiD. Only gender was identified as a factor influencing the decision for DiD or PD. Based on rating scales, a significantly higher proportion of women had depressive comorbidities at baseline.

**Conclusions:**

Demographic characteristics of the two cohorts were similar at baseline. More women chose a paper diary, and more had depression at baseline. These imbalances will be addressed in the analysis of the study as possible confounders influencing long-term treatment adherence in the digital and paper diary cohorts.

**Trial Registration:**

ClinicalTrials.gov Identifier: NCT00902135.

## Background

Multiple sclerosis (MS) is a chronic inflammatory disease of the central nervous system resulting in functional deficits due to demyelination and axonal degeneration. Disease-modifying drugs (DMD) such as interferon (IFN) beta or glatiramer acetate are the current drugs of choice for the first-line treatment of MS. As a chronic disease, MS requires long-term treatment, hence close adherence to prescribed therapy is a prerequisite for long-term benefit. Among people with chronic diseases, low adherence rates to long-term medication are a common problem, and non-adherence rates of up to 50% on average have been estimated by the World Health Organization depending on the disorder [1,2].

As a consequence, enormous costs for healthcare systems may be created by poor adherence because of relapses, prolonged duration of disease states, hospital admissions, early retirement, and secondary diseases [3,4]. Non-adherence rates for MS vary between 6 and 43% depending on factors such as type of study, definition of non-adherence, type of DMD and individual disease state [5,6]. Non-adherence of MS patients to their DMD may be defined as either termination of therapy or interruption of regular administration. Treatment adherence is influenced by a variety of factors, including factors related to the MS centre or the patient’s clinical condition, side effects, involvement in treatment decisions or way of coping [5]. Recent studies revealed that besides physical and psychological factors, depression, pain from injection, skin reactions and patient- and physician-related factors may affect adherence to medication [5,7]. Tremlett et al. identified lower education levels, increased alcohol consumption, and history of missed doses as further factors that reduce adherence [8].

The current DMDs have to be administered regularly following strict injection schedules. Missing one or more doses over a given period has been used as a definition for non-adherence [9,10]. Missing a single injection usually has no clinical relevance. Interruption of DMD treatment, however, may result in loss of efficacy and decreased treatment benefit [10]. Improvement and maintenance of adherence to DMDs is therefore of great importance in ensuring a positive influence on the course of disease in MS patients. This means that interventions intended to enhance medication adherence have to cover different aspects to achieve overall effectiveness, such as providing the patient with detailed information on treatment, realistic treatment expectations, convenient care, crisis intervention, optimized support by nursing services, and counselling [11,12].

One of the most frequent reasons for missing injections is simply forgetting to administer the medication [8,10,13,14]. Thus, measures that regularly remind the patient to administer the medication may help to improve adherence, and ultimately clinical outcome. The use of an electronic diary may be a way to address this problem. The use of electronic diaries is well established in conditions such as haemophilia and migraine [15-17], but they are not yet in widespread use by MS patients. They are also a reliable means of supporting self-monitoring, documentation of relevant symptoms or side effects, and documenting and assisting disease management.

The goal of the study described here – the “**Beta**feron® injection management: Non-interventional study on **P**DA-supported effects on **A**dherence to long-term injection **Th**erapy (BETAPATH)” study – is to compare treatment adherence to IFN beta-1b (Betaferon®) in CIS- and RRMS patients under daily-life treatment conditions using a paper diary (PD) or an electronic diary in the form of a Personal Digital Assistant (PDA, or digital diary [DiD]) with and without a reminder function. Here we report on the design of this study and the findings of baseline assessments.

## Methods

**Figure 1:**
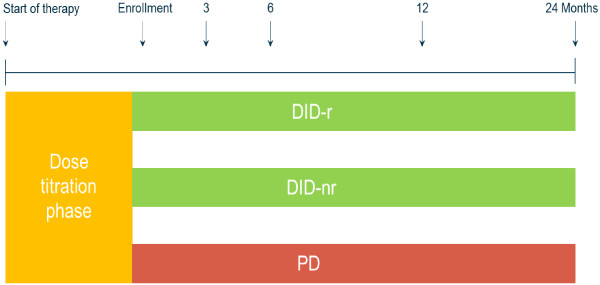
**Study design.** After completion of the dose titration phase, patients were enrolled and opted for the DiD or PD. DiD-r and DiD-nr were randomly assigned.

**Figure 2:**
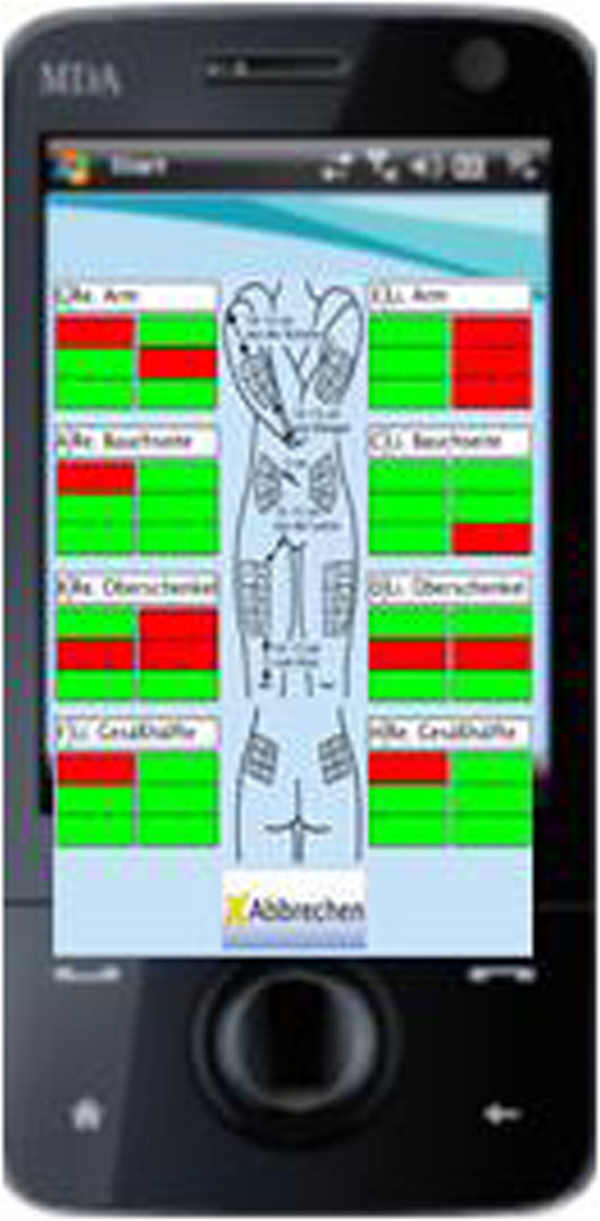
Picture of the digital diary.

BETAPATH is a national, multicentre, prospective, observational, non-interventional cohort study in MS patients in the care of hospital neurological departments and office practices in Germany. Recruitment lasted from 05/2009 to 12/2011. 700 patients with CIS or RRMS, who had started treatment with IFN beta-1b (Betaferon®), were included. The patients are being followed up for up to 2 years after the first visit (Figure 1). They were offered the use of a DiD (Figure 2) or a PD. Patients who opted for a DiD were randomly assigned a device with (DiD-r) or without (DiD-nr) an injection reminder function. All patients were asked to document their self-administration of IFN beta-1b, which is approved for administration every other day in the standard dosage (8 MIU). Deviations from this injection schedule were to be documented in the DiD or the PD. The study was approved by the Ethics Committee of the Ärztekammer Nordrhein and registered at clinicaltrials.gov (NCT00902135). All participants had to give written informed consent. Quality reviews are being conducted by an independent contract research organization (CRO) by telephone and on site-interviews twice during the study for 5% of the enrolled patients. Sites to be monitored are selected by the CRO based on a defined composite score.

Adult RRMS and CIS outpatients aged at least 18 years who had completed initial dose titration or started treatment with IFN beta-1b no longer than 3 months earlier were invited to participate. Patients were not invited to take part in the study until the decision had been taken to start them on IFN beta-1b.

The physician or nurse responsible explained to the patient how to use the DiD. A hotline for questions was also available. The DiD is a commercially available Smartphone with all standard and cost functions blocked (Figure 2). Half of the devices are equipped with an injection reminder. Devices with and without the reminder function were randomly allocated. All DiDs provide an injection scheme and document the injection history. They also make suggestions for suitable injection sites for upcoming injections, and have a diary and a help function with information on the administration of IFN beta-1b. Other injection-relevant details can also be recorded, such as side effects. The user records every injection with the date on the DiD. All entries are transmitted online to a central database maintained by an external service provider. Legal requirements with respect to confidentiality of patient data are guaranteed.

After the initial visit, follow-up visits are conducted after 3, 6, 12 and 24 months. Table 1 shows the schedule of investigations during the study.

**Table 1:** Visit schedule

	**Baseline visit**	**FU visit 1**	**FU visit 2**	**FU visit 3**	**Last visit**
	**Day 1**	**Month 3**	**Month 6**	**Month 12**	**Month 24**
Patient information and informed consent	X				
Demographics	X				
Neurological history, clinical events, disease course	X	X	X	X	X
Disability (EDSS)	X	X	X	X	X
AEs		X	X	X	X
MRI findings if available	X	X	X	X	X
Medication	X	X	X	X	X
Cognitive status, PASAT	X			X	X
Patient diary		X	X	X	X
CES-D	X	X	X	X	X
Fatigue Scale (WEIMuS)	X	X	X	X	X
FAMS	X	X	X	X	X
EQ-5D	X	X	X	X	X
Treatment Support Questionnaire				X	X

*FU visit* Follow-up visit.

*AE* Adverse event.

The primary outcome measure of adherence to the therapy with IFN beta-1b is overall continuity of treatment. All patients who discontinue treatment before week 104 for any reason specified in the case report form (CRF) e.g. an adverse event, withdrawal of informed consent, loss to follow-up, are classed as dropouts.

The secondary outcome measure of treatment adherence is the overall regularity of IFN beta-1b injections in relation to the prescribed ‘every other day’ schedule. Subjects are considered adherent to the injection schedule if they do not miss more than 5 injections every 6 months over the 2-year period. Dropouts are considered adherent to the injection schedule if they are treated for at least 6 months and are adherent to the injection schedule up to their last visit in this non-interventional study (NIS)*.* For patients who dropped out for reasons not related to adherence, e.g. loss to follow-up, death, closure of site, termination of study, adherence is assessed up to the time of the event, even if it occurs before the completion of 6 months in the study.

Other secondary outcome measures include adherence for each 6-month interval, dropout rates by dropout reason, patient satisfaction and tolerability, also with regard to the injection devices used, and clinical course, e.g. relapse rate, grade of disability. Patient-related health outcomes such as depression, cognition, fatigue and quality of life are also assessed. Safety-related variables consist of a general tolerability assessment by the physician and reports of adverse events with an assessment of causal relationship, seriousness, action taken, and outcome.

Throughout the study, patient-reported outcomes are documented by the following rating scales FAMS (Functional Assessment in MS [18], higher scores = better outcome), CES-D (Center of Epidemiologic Studies Depression Scale [19-21], higher score = more depressiveness), WEIMuS (Würzburger Erschöpfungsinventar bei MS [22], higher score = more fatigue), EQ5-D (EuroQuol-5D Health State [23], higher score = better quality of life) and EDSS (Expanded Disability Status Scale [24], higher score = more disability).

### Statistics

Descriptive analysis of the data was performed using summary statistics for categorical and quantitative (continuous) data. Continuous data were described by number of non-missing values, mean, standard deviation, minimum, median and maximum. Categorical data, including categories of continuous data, are presented in frequency tables.

Significance calculations were performed based on t-tests for continuous variables, and on χ^2^-tests or (if possible) exact Fisher tests for categorical variables. As the analyses are exploratory, significant p-values <0.05 should be interpreted as exploratory results. As BETAPATH is a NIS and analyses are based on an interim database, missing values may appear for all variables analysed and percentages for categorical variables may therefore not add up to 100%.

## Results

Between May 2009 and December 2011, 700 patients (496 women [70.9%] and 201 men [28.7%]; 3: sex missing) with CIS (35) or RRMS (662, 3 missing) were enrolled at 164 sites and had baseline documentation. Of these, 317 are using a PD and the remaining 383 are using a DiD (192 with reminder function, 191 without).

The demographic characteristics of the patient population on enrolment are summarized in Table 2. The mean age was 38.3 (SD 10.3) years, and the mean duration of disease since diagnosis of MS 3.6 (SD 5.9) years. There were no significant differences between the cohorts (PD, DiD) with respect to duration of illness, relapse rate, grade of disability (EDSS), educational level and social status. For age and body weight, there were small but significant differences. While exactly 50% of the women chose the DiD, a significantly larger proportion of men (66.7 vs 33.3%, p<0.001) opted for the DiD. Most women and men were living with a partner on enrolment, but more men than women living alone are using the DiD (81.8 vs 53.1%, p=0.001). The patients living with a partner and using a PD are older than those using a DiD (40.1 vs 37.8 years, p=0.007), but there was no significant difference for the group living alone.

**Table 2:** Demographic characteristics

	**Total**	**PD**	**DiD**	**p value**
No. of patients	700	317	383	
Age [years] - mean (SD)	38.3 (10.3)	39.8 (10.6)	37.0 (9.9)	^1)^ p<0.001
Body weight [kg] - mean (SD)	73.6 (15.5)	71.5 (13.8)	75.3 (16.5)	^1)^ p<0.001
Men - n (%)	201 (28.7)	67 (21.1)	134 (35.0)	^2)^ p<0.001
Women - n (%)	496 (70.9)	248 (78.2)	248 (64.8)	
Diagnosis [years] before - mean (SD)	3.6 (5.9)	3.7 (5.8)	3.5 (6.0)	^1)^ p=0.668
Relapse rate last two years - mean (SD)	1.6 (1.3)	1.7 (1.2)	1.6 (1.3)	^1)^ p=0.357
EDSS - mean (SD)	2.0 (1.4)	2.1 (1.5)	2.0 (1.4)	^1)^ p=0.149
Diagnosis CIS - n (%)	35 (5.0)	11 (3.5)	24 (6.3)	^2)^ p=0.117
Diagnosis RRMS - n (%)	662 (94.6)	304 (95.9)	358 (93.5)	
Living with partner - n (%)	555 (79.3)	261 (82.3)	294 (76.8)	^2)^ p=0.059
Living alone - n (%)	142 (20.3)	54 (17.0)	88 (23.0)	
Educational status				^2)^ p=0.264
- Elementary school - n (%)	151 (21.6)	75 (23.7)	76 (19.8)	
- Secondary school - n (%)	353 (50.4)	164 (51.7)	189 (49.3)	
- High school - n (%)	121 (17.3)	50 (15.8)	71 (18.5)	
- University - n (%)	70 (10.0)	26 (8.2)	44 (11.5)	

^1)^ t-test / ^2)^ Fisher test.

Of the 700 patients enrolled, 498 (343 women, 155 men) were treatment-naïve for DMDs. Of these 498, 223 opted for a PD and 275 for the DiD. Amongst the latter, 104 were men (37.8%), while there are only 51 men in the PD group (22.9%, p<0.001). The patients with pretreatment with any MS therapy (197, 46 men, 151 women) were significantly older than those without pretreatment (Table 3). In both the untreated and the pretreated groups, the PD users are significantly older than the DiD users (Table 3).

**Table 3:** Age of study cohorts by previous DMD treatment

**Previous treatment**	**Total**	**PD**	**DiD**	**p-value**
**No**				
n patients	498	223	275	
age [years] - mean (SD)	37.3 (10.4)	38.6 (10.6)	36.3 (10.1)	0.011
**Yes**				
n patients	197	91	106	
age [years] - mean (SD)	40.6 (9.7)	42.5 (10.1)	39.0 (9.2)	0.013

p<0.001 for difference in age (previous treatment no vs. yes in total population).

The duration of disease in the pretreated group was longer: MS was diagnosed 7.1 years (SD 6.4) before enrolment, compared to 2.2 years (SD 5.0) in the treatment-naïve group. More than 60% of the patients had a history of MS of less than 2 years, which is reflected in the high proportion of patients without DMD pretreatment. The mean EDSS score for all patients enrolled was 2.0 (SD 1.4), with no difference between the PD and DiD groups.

Patient-reported outcomes assessed by the different rating scales are summarized in Table 4. The mean FAMS total and the FAMS-TOI scores were slightly but significantly higher in the DiD (134.8 and 111.5 respectively) than the PD group (129.3 and 106.8), indicating better MS-related quality of life in the DiD group. The total scores of the WEIMuS ranged around 20 and the mean values for the VAS of the EQ-5D were about 74, reflecting a low level of fatigue and a relatively high level of health-related quality of life. No significant differences were found between the DiD and PD groups with these two instruments.

**Table 4:** Results of patient-reported outcomes of rating scales

**Rating scale score**	**Total**		**PD**		**DiD**		**p-value**
	**mean**	**(SD)**	**mean**	**(SD)**	**mean**	**(SD)**	
FAMS total score	132.4 (29.4)		129.3 (29.5)		134.8 (29.1)		0.027
FAMS-TOI	109.5 (24.5)		106.8 (24.6)		111.5 (24.4)		0.030
CES-D score	13.2 (9.8)		14.1 (10.2)		12.4 (9.4)		0.040
WEIMus total score	20.4 (17.3)		21.2 (17.5)		19.8 (17.2)		ns
EQ-5D VAS	74.3 (18.4)		73.0 (18.0)		75.3 (18.7)		ns

*FAMS* Functional assessment in MS.

*FAMS-TOI* FAMS Trial Outcome Index.

*CES-D* Center for Epidemiologic Studies Depression Scale.

*WEIMuS* Würzburger Erschöpfungsinventar bei MS.

*EQ5-D VAS* EuroQuol 5-D Visual Analogue Scale.

*ns* non significant.

**Figure 3:**
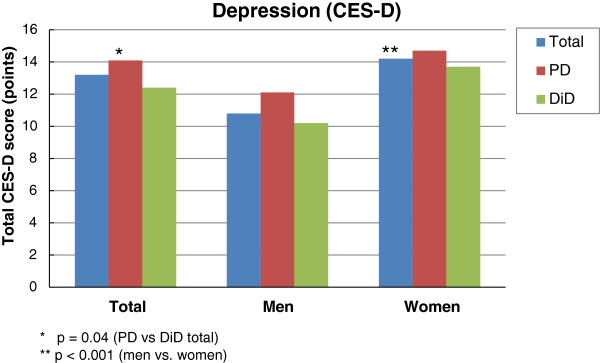
Total CES-D scores stratified by sex of the total population and the PD and DiD cohorts.

With respect to depressiveness rated on the CES-D scale, the total score in the PD group was slightly but significantly higher than in the DiD group. The mean CES-D score for the total study sample was 13.2 (SD 9.8, range 0–48) and for the PD and DiD subgroups, 14.1 (SD 10.2, range 0–48) and 12.4 (SD 9.4, range 0–43) respectively (Figure 3). Women had a significantly higher mean value for the CES-D total score (Figure 3). Within the gender subgroups, however, no significant differences between the PD and DiD groups were found (data not shown). The cut-off for the presence of depressive symptoms is ≥16. 62.2% of patients had a score between 0 and 15 (no depressive symptoms) and 31.9% had a score ≥ 16. Significantly more women had a score ≥ 16 (p<0.001). Amongst all patients, 66 (11.1%) had scores of 16–20 (slightly depressed), 53 (8.9%) 21–25 (moderately depressed), and 70 (11.8%) ≥26 (severely depressed) (Table 5). In these three groups there was a significant difference between the proportions of women and men (p=0.001), in particular with more women than men among the moderately (10.6 (n=45) vs 4.8% (8)) and severely depressed patients (13.9 (59) vs 6.5% (11)). No significant differences were found for grouped CES-D scores between the PD and DiD groups (Table 5). A higher (14.5 vs 12.7; p=0.064) mean CES-D total score was found for the pretreated subjects. A higher mean total score (14.1 vs 11.7; p=0.016) for the PD than the DiD group was found for patients without DMD pretreatment, while no difference was observed in pretreated patients.

**Table 5:** Grouped CES-D total score at baseline

**CES-D**	**Total**		**PD**		**DiD**		
	**n**	**(%)**	**n**	**(%)**	**n**	**(%)**	**p-value**
Total	593 (100.0)		262 (100.0)		331 (100.0)		ns
Missing	35 (5.9)		16 (6.1)		19 (5.7)		
0–15	369 (62.2)		153 (58.4)		216 (65.3)		
≥16	189 (31.9)		93 (35.5)		96 (29.0)		
16–20	66 (11.1)		30 (11.5)		36 (10.9)		
21–25	53 (8.9)		28 (10.7)		25 (7.6)		
≥26	70 (11.8)		35 (13.4)		35 (10.6)		

≥16 defined as cut off for the presence of depressive symptoms.

16-20 slightly depressed; 21-25 moderately depressed, ≥26 severely depressed.

*ns* = non significant.

**Figure 4:**
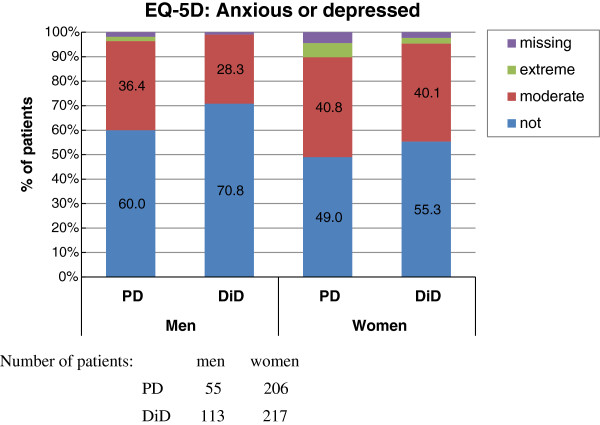
State of anxiety or depression assessed using the EQ-5D in men and women using a PD or DiD.

In line with these results, the anxiety/depression dimension of the EQ-5D health state showed that about 37.6% of the patients were moderately anxious or depressed, 3.0% extremely so, and that 56.7% were not anxious or depressed. The proportion of patients without anxiety/depression was significantly higher in the DiD group than the PD group (Table 6) and in men (Figure 4). This difference was even more obvious in patients without pretreatment: 63.2% (n=158) of the DiD group had no depression or anxiety and 50.8% (n=95) in the PD group (p=0.008).

**Table 6:** EQ-5D Health State: anxiety/depression

**EQ-5D**	**Total**		**PD**		**DiD**		**p-value**
	**n**	**(%)**	**n**	**(%)**	**n**	**(%)**	
Total	593 (100.0)		262 (100.0)		331 (100.0)		0.016
Missing	16 (2.7)		10 (3.8)		6 (1.8)		
Not anxious or depressed	336 (56.7)		135 (51.5)		201 (60.7)		
Moderately anxious or depressed	223 (37.6)		104 (39.7)		119 (36.0)		
Extremely anxious or depressed	18 (3.0)		13 (5.0)		5 (1.5)		

## Discussion

Despite many attempts to improve and facilitate the administration of DMDs for MS patients, e.g. by assistance from a special MS nurse and auto-injectors, non-adherence to a prescribed therapy and treatment schedule is still a problem for the long-term treatment of MS patients. The study described here is evaluating the influence of a digital diary (with and without a reminder function) on treatment adherence to IFN beta-1b in routine clinical practice. In keeping with the non-interventional nature of the study, patients decided themselves whether to use a digital or paper diary to record treatment administration. Careful comparison of baseline characteristics of both study cohorts will therefore be necessary before analysis of adherence rates. This will allow for identification and consideration of possible confounding factors. A special feature of our study is the randomized distribution of the DiDs with and without a reminder function. This design will allow the direct assessment of effects of the reminder function on the overall dropout rate and the regularity of the injection schedule by comparing the findings in the two study cohorts. Thus, the present study may result in a further option in addition to the approaches using injection devices and nurse support services discussed recently [25].

Our study is a prospective trial with an observation period of up to two years and a study population of 700 patients. It is one of the largest single studies focusing on long-term treatment adherence in MS using a digital diary. Findings for the characteristics of the study population at baseline have already been evaluated. Demographic and baseline findings for educational level, living status, duration of disease since start of symptoms, number of relapses, EDSS, impairment of upper extremities, visual impairment, and cognitive assessment did not differ between the groups using the digital diary (DiD) and the paper diary (PD). An imbalance in gender distribution was, however, found showing that male gender was significantly associated with usage of the DiD, as well as younger age. We found nothing in the literature to support a greater preference amongst men for technical devices, and the demographics of our study population do not explain this gender effect. Several studies have focused on gender differences in the use of computers in general [26], and one study on the use of patient’s electronic personal health records by physicians [27]. These studies showed that women are less willing to use computers and that female physicians are less willing to use electronic tools.

Analysis of psychometric test results at baseline revealed no difference between the study cohorts for fatigue measured on the WEIMuS scale or for health-related quality of life assessed on the VAS of the EQ-5D. The mean scores on the WEIMuS scale of around 20 (maximum 68) are similar to values obtained in healthy control persons [22], and hence indicate a low level of fatigue. In line with this, values obtained on the EQ-5D-VAS of approximately 74 (maximum 100) indicate a high mean quality of life amongst the study population. This is further supported by the mean total scores obtained on the FAMS rating scale of 129 and 134 for the PD and DiD groups respectively (maximum 176). Taken together, these results suggest that a majority of patients enjoy a high degree of health-related quality of life, which may be related to age and the relatively short time since diagnosis of MS of the study population.

Higher values for the PD group were found for the scores on EQ-5D health state anxiety subscale and the CES-D depression rating scale. When the total study cohort was stratified by gender a higher degree of depressive symptoms was found in women. However, after further stratification of the gender cohorts into DiD and PD subgroups the CES-D total score was no longer higher in the PD group suggesting that the association of depression and preference for PD usage can mainly be explained by female gender and not by severity of depression. The mean CES-D total scores for the whole study population and for men and women ranged between 10.8 and 14.2, and were therefore below the cut-off of 15, below which no relevant depressive comorbidities are expected. About 32% of the total study population, however, had a CES-D total score of ≥ 16, suggesting that a substantial part of these patients will show some depressive morbidity during the study [28]. This result is in line with previous evaluations using the CES-D in male and female MS patients [29], but much lower than in an epidemiological study of a large community sample in which 42% of the cohort had clinically significant symptoms [30]. However, the patients in the latter study had a much higher mean age (49.3 years) and a much longer mean duration of illness (12.5 years). Correspondingly, the mean disability status of this cohort was higher than in the present population (5.6 for women and 6.1 for men vs 2.0). Since the severity of illness as reflected by the grade of disability has been identified as the greatest factor associated with depressive symptoms [30], this might be an explanation for the different ratios of depressed and non-depressed subjects.

The findings presented here show an association between depressive symptoms and female gender. This is not surprising, as depression is about twice as common in women than in men in the general population [31]. It has been shown that patients with symptoms of depression are more likely to discontinue their DMD and that suitable treatment of their depression may increase adherence [32].

## Conclusions

Amongst a large sample of MS patients enrolled into the non-interventional BETAPATH study, half of the women and two-thirds of the men chose to document their treatment using an electronic diary and the remainder chose a traditional paper diary. Demographic characteristics of the two cohorts were similar at baseline, except with regard to gender, which emerged as the main distinguishing factor. A further potential confounder was depression at baseline, which was disproportionately higher in females. Both factors must be taken into account in the analysis of findings.

## Trial status

This NIS is currently in the last year of follow-up observation.

## Competing interests

UKZ has received speaker honoraria and travel grants from Bayer Pharma, Aventis Pharma, TEVA Pharma, Merck-Serono Pharma and Biogen-Idec Pharma. KH: No competing interests. VL has received speaker honoraria and research support from Allergan, Bayer, BiogenIdec, Genzyme, GSK, MSD, Novartis, Pfizer, Roche, St. Jude. UBS and TG are full-time employees of Bayer Vital GmbH, Leverkusen.

## Authors’ contributions

UKZ, UBS, TG and VL participated to equal extents in the design and management of the study; KH is involved in defining the statistical plan and performing the statistical analyses. All authors contributed to drafting the manuscript and read and approved the final version.

## Pre-publication history

The pre-publication history for this paper can be accessed here:

http://www.biomedcentral.com/1471-2377/13/117/prepub
